# Novel Approaches in the Valorization of Agricultural Wastes and Their Applications

**DOI:** 10.1021/acs.jafc.1c07104

**Published:** 2022-02-23

**Authors:** Esra Capanoglu, Elifsu Nemli, Francisco Tomas-Barberan

**Affiliations:** † Department of Food Engineering, Faculty of Chemical and Metallurgical Engineering, Istanbul Technical University, 34469 Maslak, Istanbul, Turkey; ‡ Quality, Safety, and Bioactivity of Plant Foods, CEBAS-CSIC, 30100 Murcia, Spain

**Keywords:** agricultural food waste, agricultural byproduct, waste management, waste valorization, nanotechnology, biotechnology, food application

## Abstract

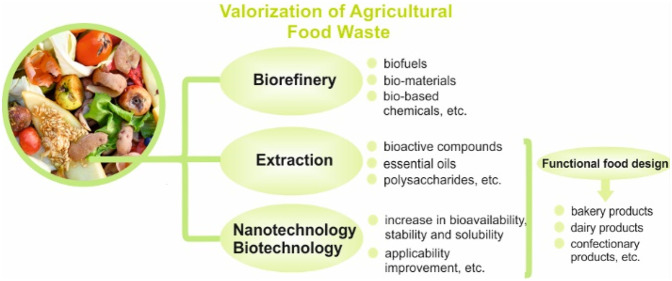

Worldwide, a huge amount of agricultural food wastes and byproducts containing valuable bioactive compounds are generated, especially throughout the entire supply chain. Minimizing food wastes and byproducts is the first option to avoid environmental problems, and to help the economy and the society. Although many countries implement policies to reduce food wastes and byproducts, and different management methods are available to utilize agricultural food wastes, they are still produced annually. Nanotechnological and biotechnological approaches are recently used as novel and green applications to valorize agricultural food wastes and improve their stability and applicability. In this Review, these approaches are covered in detail with given examples. Another valorization way of consumable food waste is using it for functional food production. This Review focuses on specific examples of functional foods with food waste as an ingredient. In addition, the problems and limitations of waste management and valorization methods are investigated, considering future perspectives.

## 1 Introduction

Agricultural and food wastes constitute a significant problem worldwide, because of the adverse effects that agricultural waste has on the environment, economy, and society. Various scientific studies focus on the management of food waste. Food loss is defined as a decrease in food quantity and quality that occurs due to decisions and actions of the suppliers in the food chain.^1^ On the other hand, food waste is defined as a decrease in food quantity and quality due to the decisions and actions of retailers, food service providers, and consumers.^1^ The main reason for food waste and loss is rapid global population growth and food consumption behavior.^2^ The environmental effects occurring during food production are increased by food loss in the food system.^3^ The total global food loss and waste, ∼1.3 billion tons annually, corresponds to one-third of the food produced for human consumption. The food waste is made by using nearly 30% of the agricultural land area in the world.^4^ The Food Waste Index Report stated that ∼931 million tons of food waste, including 61% household, 26% food service, and 13% retail waste, were generated in 2019.^5^ The generation of food waste, primarily from the household, is related to purchasing power of consumers determined by the income levels of countries.^3,5^ Latin America and Europe have the highest consumer waste with 200 and 180 kg per capita per year, respectively. Each of North America and Oceania, North Africa, and West and Central Asia have 175 kg consumer waste per capita per year. They are followed by the consumer waste of industrialized Asia (155 kg), sub-Saharan Africa (150 kg), and south and southeast Asia (110 kg) per capita per year.^2^ In 2016, Central and Southern Asia, Northern America, and Europe were identified as the first three regions with the highest food loss, considering the entire supply chain from post-harvest to distribution.^1^ The approximate food waste is 307 g per capita per day for high-income countries, corresponding to twice that of the upper-middle income countries.^3^ Food waste is obtained primarily at later stages of the supply chain in industrialized countries. However, because of lacking financial and technical properties during harvesting, storage, and cooling, most of the waste in developing countries is obtained at early stages in the food supply chain.^6^

The residues of raw agricultural products constitute the agricultural wastes.^7^ Such residues include manure and animal carcasses (animal waste); corn stalks, sugar cane bagasse, drops and culls from fruits and vegetables, pruning (crop waste); pesticides, insecticides, and herbicides (hazardous and toxic agricultural waste); and food processing waste obtained during growing and processing in liquid, slurry, or solid forms. The main food groups contributing to nutrient and food waste or loss are cereals and pulses, fruits and vegetables, meat and animal products, roots, tubers, and oil-bearing crops.^1,3,6^ Among these food groups, roots, tubers, and oil-bearing crops with almost 26% and fruits and vegetables with nearly 22% are the first two groups, based on food loss.^1^ The cereals, root crops/fruits/vegetables, and oilseeds compose the global annual food waste with 30%, 40–50%, and 20%, respectively.^6^ Without considering the agricultural food losses, food waste is produced within the food chain, including 42% of households, 38% of food processing, and 20% of other processes. Considering the food supply chain, the beverage industry produces food waste with 26%. It is followed by the dairy and ice cream industry (21.3%), fruit and vegetable production and preservation (14.8%), the manufacture of grain and starch products (12.9%), meat production, processing, and conservation (8%), the manufacture of vegetable and animal oils and fats (3.9%), the production and preservation of fish and fish products (0.4%), and the manufacture of other food products (12.7%).^8^ The animal-derived food wastes contain fats, lard, blood, internal organs of farm animals, offal, head, tails, scales, shells of marine animals, and dairy products such as cheese whey, curd, and milk sludge. The wastes of vegetables, fruits, cereals, roots and tubers, oil crops, and pulses consist of peels, stems, seeds, shells, bran, germs, cull, pomace, pulp, and other residues obtained from processing.^8,9^ The estimated wastes arising from food supply chains based on geographical locations mentioned in several studies can be summarized with some examples given for annual waste amount in tons as the following: 50 000–100 000 vegetable oil waste in the UK, 4 000 000 tomato pomace in Europe, 57 000 wheat straw in the USA, 40 000–45 000 cereal waste in Europe, 700 orange peel in the USA, 700 grape pomace in France, 2 881 500 olive pomace worldwide, 3 000 000–4 200 000 apple pomace worldwide, and 70–140 potato peel worldwide.^10^

This paper emphasizes the significance of food wastes and their effects on nutrition, environment, economy, and management systems. On the other hand, different methods used to valorize agricultural food wastes and improve their stability and applicability are reviewed. Besides, some specific examples of applying ingredients obtained from waste materials in functional foods are provided. Finally, the limitations and problems encountered during the management and application of these wastes, together with future perspectives, are mentioned.

## 2 Significance of Food Wastes from Nutritional, Environmental, and Economical Points of View

Food waste is considered a significant source of complex carbohydrates, proteins, lipids, and phytochemicals, because of its high contents of polysaccharides, dietary fibers, oils, vitamins, phenolics, carotenoids, and other pigments. Therefore, the potential health benefits of wasted foods rely on their high contents of biologically active compounds.^10,11^ Food waste has social impacts associated with nutrient loss and world hunger. The wasted foods can theoretically fill nutritional gaps for millions of people. For example, the annual amount of food loss and waste can provide a diet of 2100 kcal per day for 2 billion people. According to FAO records, this potential of food loss and waste is crucial since 690 million people were estimated to be sufffering from hunger in 2019. This number has increased dramatically during the COVID-19 pandemic, and it is expected to increase further more.^5,12^ Food waste of the U.S. food supply in 2012 at the retail and consumer levels consists of 33 g of protein, 5.9 g of dietary fiber, 1.7 μg of vitamin D, 286 mg of calcium, 880 mg of potassium, and 1217 kcal per capita per day.^13^ Also, it has been reported that the wasted calcium, choline, riboflavin, zinc, and vitamin B12 especially arise from the loss of meat, dairy, and eggs.^3^ The daily food waste per person corresponds to the food of 795–840 kcal. And, carotenoids have the highest value with 31%, and vitamin D has the lowest value with 25% within all wasted nutrients.^14^ Because of these wasted nutritious foods, food waste reduction can provide more available nutrients for human consumption.^12^ If the necessary precautions for food waste reduction are not taken, and food consumption and production are not planned, the World in 2050, with a population of more than nine billion, will need 60% more food, which equals at least 2 billion tons. The predictions show that the food gap in 2050 can be reduced by ∼20% via decreasing the global food waste by half.^15^

**Figure 1:**
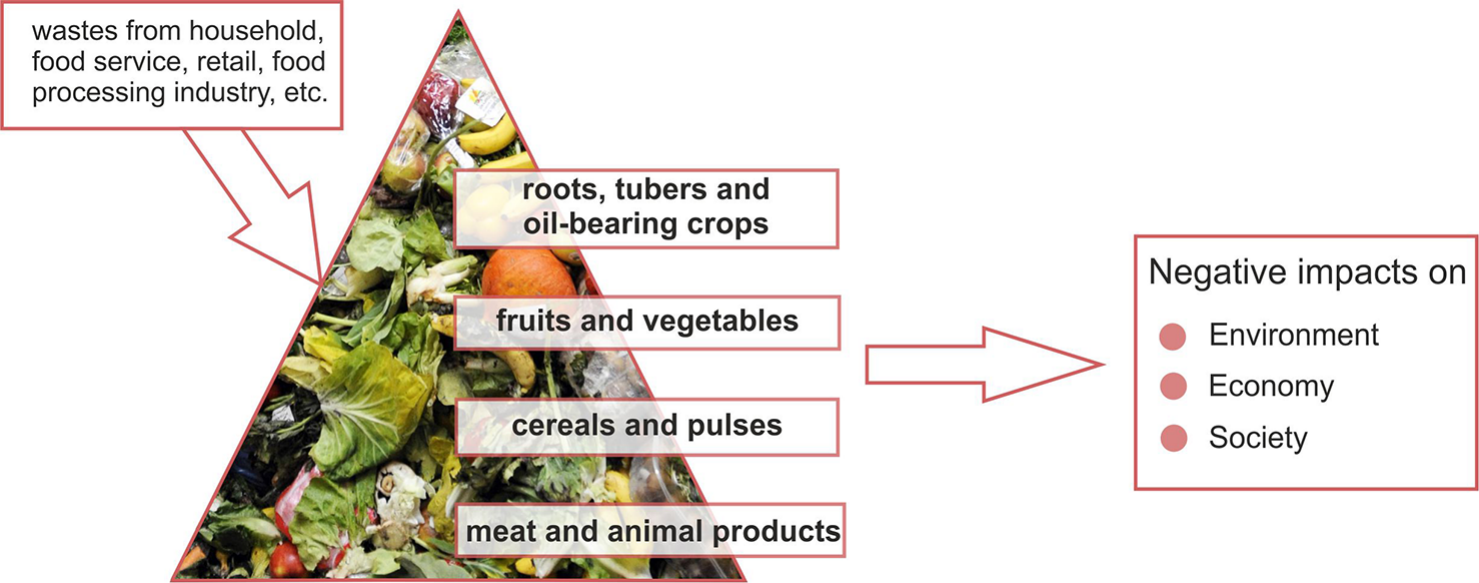
Impacts of agricultural food wastes and byproducts.

Food waste has environmental and economic impacts right along with its social impacts (Figure 1). Food waste has devastating effects on climate change.^16^ The estimated carbon footprint contribution of food waste to greenhouse gas (GHG) emissions equals ∼3.3 billion tons of CO_2_ accumulation in the atmosphere per year.^17^ The food consumption and waste generation trends of 17 110 family members in China, a highly populated country, have been investigated based on the household’s carbon, water, and ecological footprint quantification. The annual consumption of food at home (415 kg) resulted with 1080 kg CO_2_ eq of carbon, 673 m^3^ of water, and 4956 gm^2^ of ecological footprints, whereas the annual wastage of food at home (16 kg) causes 40 kg CO_2_ eq of carbon, 18 m^3^ of water, and 173 gm^2^ of ecological footprints.^18^ Food waste can also create many other environmental problems, since it is disposed without any appropriate pretreatment by landfill or incineration in the dumping sites.^2^ Some environmental issues causing respiration difficulties for living organisms and air pollution due to dioxin, ash, and flue gas released to the atmosphere are created by the incineration of food wastes.^2,17^ As is the case with the incineration of food waste, toxic byproducts contaminate groundwater and cause corrosive gases, such as methane and hydrogen sulfide, to be generated by food waste landfilling.^2^ The landfilling of food waste reduces its energy content. The energy loss at landfill sites is equivalent to 43% of the delivered energy for food preparation in the U.S. and more than 100% of the current annual renewable energy demand of industries in the U.K.^16^

Another important issue related to food waste and loss is economic problems. The approximate global cost of food waste equals 1000 billion dollars annually. This number can increase up to 2600 billion dollars when ignored environmental costs are taken into account.^19^ FAO reported that the cost of total food waste amount in 2007 was almost 750 billion dollars, which is approximately equal to the gross domestic product (GDP) of Turkey and Switzerland in 2011. Vegetables primarily contribute to the economic cost of food waste and loss with 23%, and also meat, fruits, and cereals contribute to the total cost with the percentages of 21%, 19%, and 18%, respectively.^4^ Annually, more than 55 million metric tons of avoidable food wastes are produced in the U.S., corresponding to almost 29% of the annual production. The cost of these wasted foods equals 198 billion dollars.^20^ At least 18.6 billion dollars are lost in the U.K. with 8.3 billion metric tons of annual household food waste.^20,21^ Therefore, since food waste reduction can help providing sufficient food for the increasing population globally, it is a critical issue in terms of economy, environment, and society.^22^

## 3 Food Waste Management

The first option to manage food waste is to prevent waste generation. Reusing and recycling are considered as secondary options in food waste management.^23,24^ Other methods, such as reduce–reuse–recycle, extended producer responsibility, and sustainable management to reduce the wasted food, have been developed to manage the food wastage.^23^ Food losses can also be prevented by local investments, educations, ensuring the cold chain, improving packaging and market facilities in low-income countries. For high-income countries, enhancing communication in the supply chain, improving purchase/consumption planning, and awareness of the best-before-dates can be options for food loss prevention.^8,25^ To manage or reduce food waste, the countries apply several policies for individuals, organizations, and businesses, depending on their consumer behaviors, income levels, and development levels.^22,26^ The European Union Waste Directive supports its members for preparing required programs to manage and reduce their food waste by 30% by 2025. Italy and France apply the same programs countrywide, whereas Austria, Czech Republic, Poland, Netherlands, Sweden, and Scotland, apply their programs at the municipal level.^24^ Also, in the U.S., Food Waste Challenge (FWC) and EPA Food Recovery Challenge (FRC) are preferred as waste reduction recognition programs.^22^

Although the best options are prevention or reduction of the food waste, according to the food waste management methods, the valorization of food waste can be considered one of the best methods when prevention or reduction is not possible. Valorization refers to the diversion of former food waste to food and feed products. It also includes converting food waste to extracted food and feed ingredients, considering food waste’s quality, robustness, and composition. The conversion of food waste requires evaluating the market conditions regarding technological feasibility, economic viability, legislative reportability, and environmental sustainability and utility.^27^ Food wastes, which potentially contain carbohydrates, proteins, lipids, and nutraceuticals, are composed of different constituents.^10,17^ Carbohydrates are mostly derived from food waste containing rice and vegetables, whereas proteins and lipids mostly derive from meat and egg wastes.^17^ The food waste has started to be assessed as valuable biomass that can be utilized as profitable products instead of an uncontrollable discard. Since food waste is renewable and inexpensive, it can be beneficial for obtaining energy, biofuels, enzymes, antioxidant extracts, novel biodegradable materials, and other commercial products.^9^ Although it is stated that the governments in the European Union (EU) will struggle with the valorization of food waste at a larger scale within the next decades, the EU defined the concept of biowaste valorization to obtain more valuable products in 2010. Currently, landfilling, composting, and incineration are three popular choices for most food waste produced in the EU. However, the increase in power purchasing and the worsening of food management have led to the rise in food waste generation in recent decades, making food valorization a crucial subject for society.^28^

## 4 Novel Approaches for the Valorization of Agricultural Food Wastes/Byproducts

### 4.1 Valorization Methods of Agricultural Food Wastes/Byproducts

The value-added products, which are fine chemicals, nutraceuticals, antioxidants, bioactives, biopolymers, biopeptides, antibiotics, industrial enzymes, bionanocomposites, single-cell proteins, polysaccharides, activated carbon adsorbent, chitosan, corrosion inhibitors, organic acids, pigments, sugars, wax esters, and xanthan gum, can be recovered by using food wastes as a substrate.^29,30^ The conventional treatment of food waste such as landfilling and incineration leads to environmental, economic, and social problems. Thus, several available valorization methods, which are more sustainable and profitable to manage food waste, arise as alternative options to obtain the value-added products mentioned above. Also, special chemicals refined from food waste, varying from solvent to antioxidant materials, are essential for nutraceutical and biomaterial applications.^30^ The combined methods, including biochemical, chemical, and physical steps, should be applied to separate the potentially marketable compounds found in food wastes and byproducts to selectively extract and modify the preferred components and change them to higher-value food products and additives. These methods should be applied carefully to avoid microbiological hazards and ensure that the final products are suitable for consumers’ taste and produced by following the food regulations.^8^

Only the effective utilization of renewable resources and the exploitation of renewable carbon can replace fossil resources to produce chemicals, materials, polymers, fuels, and energy. The industrial development will be sustainable through specialty product extraction, conversion by green chemical or biotechnological processes, integrated biorefining, industrial symbiosis, cascade processing, and on-site processing of seasonal waste streams, obtained by the effective exploitation of agricultural and forestry residues, aquatic biomass, and different waste streams.^31^ The coproducts, which are not appropriate for food exploitation, should be utilized as energy sources after the application of fermentation, biogas production, and composting, indicating that an integrated biorefinery approach can provide the valorization of food waste for bioactive molecule production for pharmaceutical, cosmetic, food, and nonfood applications.^8^

#### 4.1.1 Biorefinery for Agricultural Food Wastes/Byproducts

The effective valorization of byproducts obtained during biomass production, such as agricultural residues, food processing waste, and food residuals, can contribute to the global bioeconomy.^32^ The concept of biorefinery, rapidly accepted as a sustainable alternative by the scientific community, involves energy and commercial production by recycling food waste.^33^ The biotechnological techniques, including anaerobic digestion, fermentation, and composting, transform the abundant and low-cost waste biomass into biorefinery products such as biofuels and biomass biofertilizers, bioplastics, and secondary chemicals.^32−34^ In addition, these biotechnological techniques can convert the agro-food wastes into efficient biobased adsorbents used in the bioremediation of various pollutants found in wastewaters.^33^ The public perception of food waste will be changed by utilizing the food wastes more in chemical synthesis to create a closed-loop economy by providing a renewable supply chain. For example, a recent provisional agreement on the renewable energy policy, which is also targeting to obligate the development of waste-derived biofuels, is done by the EU. The agreement defining a biorefinery based on food wastes is planned to have a crucial role in contributing to a more sustainable and greener society in the future.^30^ Consequently, food waste management with the concept of biorefinery has positive environmental effects, because of less greenhouse gas emissions, environmental burden reduction of their disposal, and being more independent about the use of fossil-based sources for fuel generation.^33,34^

##### Biofuel

Biofuel, which can be in solid, liquid, and gaseous forms, is defined as the energy originated from biomass and refined products of biomass, consisting of bioethanol, biodiesel, biokerosene, natural gas, etc. From the beginning of human civilization, biofuel has been widely used in daily human activities like cooking, lighting, and heating.^35^ The production of biofuel, as an alternative fuel, is increasingly supported worldwide, because of the problems regarding the production and permanence of petroleum and coal-based fuels.^36^ Nowadays, countries are working on the utilization of their food wastes as fuels. For example, a project including Nordic countries focuses on the policies to increase the use of food wastes and the investigation of new technologies to transform their wastes into transport fuels. Denmark, Finland, and Sweden are currently using their food wastes such as fruit andvegetable wastes, animal-based wastes, bakery wastes, and biowastes from households, agricultural byproducts, industrial and commercial origins in biodiesel, bioethanol, and biogas production.^37^ Some of the most common biofuels and their applications are summarized in the following paragraphs.

Biodiesel, which is fatty acid methyl ester, is produced from several plant oils, including soybean, rapeseed, and canola, by direct or indirect transesterification.^2,36^ The study of Karmee and Lin exemplified the use of low-cost food waste in biodiesel production.^36^ They obtained the lipids by the fungal hydrolysis of food wastes, and these lipids are transesterified to produce biodiesel. Biogas like biomethane/hydrogen/hythane, another biofuel and renewable energy source, is obtained by anaerobic digestion of agro-food biomass residuals, considering the renewable energy legislation of the EU.^2,38^ However, some components in biogas, such as hydrogen sulfide,carbon dioxide, nitrogen, hydrogen, oxygen, and water vapor, cause a decrease in calorific value compared to natural gas. Some physicochemical and biological technologies include cleaning and upgrading techniques to evaluate the raw biogas quality. Biological methods, cryogenic separation, hydrate separation, membrane enrichment, in situ upgrading, multistage, and high-pressurized anaerobic digestion can be given as examples of advanced modern biogas upgrading techniques.^39^ As an example of biogas production from food waste, a study focused on improving the anaerobic digestion process used in biogas production.^40^ They concluded that the practical usage of ultrasound during the pretreatment of food wastes and the anaerobic digestion increase the yield of biogas within a shorter time range. Recently, an innovative solid-state microanaerobic digestion process has been developed to valorize food waste by degrading them. This technology makes anaerobic digestion a compact process that requires low water and energy. A quantity of 143 L/kg methane was produced via the solid-state microanaerobic digestion process, which is planned to be improved to increase its applicability and optimize its process conditions.^41^ Another popular biofuel is bioalcohol, which is considered as an emerging alternative liquid fuel, because of its petroleum-like characteristics.^33^ It represents the most commercialized transportation fuel, which achieves carbon neutrality and is compatible with an internal combustion engine.^42^ Ethanol is produced by the microbial fermentation of various feedstocks, including potatoes, molasses, corn, stover, wheat, sugar cane, bagasse, sugar beet, grain, switchgrass, barley, and many other carbohydrate-rich sources, and is the most common bioalcohol.^33,36^ In addition, fast food wastes, as a good source of carbohydrates, were used for bioethanol production. For instance, ethanol was produced by enzymatic hydrolysis with α-amylase and fermentation process from the waste of pizza with the highest yield of 0.292 g/g waste of pizza^43^ and waste of hamburger with the highest output of 0.271 g/g waste of hamburger.^44^ Biochar, a carbon-rich biofuel, is produced with a charring (or pyrolysis) process, by heating the biomass above 250 °C under limited or no air conditions.^45^ It is used as a renewable carbon material in many areas, especially soil amendment and environmental management.^30,45^ For instance, along with the benefits of biochar in mediating soil acidity, water holding capacity, cation exchange capacity, and nutrient retention, it is considered a suitable electrode in supercapacitors, which are used within green energy storage devices.^30^

##### Valuable Biomaterials

Biopolymers, bioplastics, biofertilizers, enzymes, organic acids, single-cell protein (microbial biomass) are also obtained from agricultural food wastes/byproducts with the application of different treatments like fermentation and composting. These valuable biomaterials are used in the cosmetics, pharmaceutical, chemical, food, and beverage industries.^33^ Enzymes, which are significant ingredients for different products and processes, have a massive significance in the industry, since they exhibit specificity against substrate and product, moderate reaction conditions, formation of byproducts in a minimum amount, and high yield. The raw material costs are responsible for up to 30% of the total production cost of enzymes.^46^ Therefore, the usage of food wastes and byproducts is a good option for reducing the raw material costs for the production of enzymes. It also reduces the waste amount and prevents its negative impacts on the environment. There are several studies about the recovery of enzymes from food wastes and byproducts in the literature. For example, α-amylase from coffee wastes by solid-state fermentation with a fungal strain of *Neurospora crassa* CFR 308,^47^ glucoamylase from food waste by submerged fermentation with *Aspergillus niger* UV-60,^48^ and lipase from melon wastes by solid-state fermentation with *Bacillus coagulans*,^49^ were recovered. Lactic, succinic, citric, 3-hydroxy propionic, acetic, and butyric acids are among the organic acids produced from food wastes, and organic acid production by acidogenesis is influenced by the composition of the food waste.^2^ In the study of Kim et al., kimchi cabbage waste was used in organic acid production with lactic acid bacteria. The results showed that organic acids including 12.1 and 12.7 g/L lactic acid, 7.4 and 7.1 g/L fumaric acid, 4.5 and 4.6 g/L acetic acid from the waste of kimchi cabbage with *Lactobacillus sakei* WiKim31 and *L*. *curvatus* WiKim38, respectively, were obtained by the simultaneous application of saccharification and fermentation for 48 h.^50^ Also, in another study, 47.3 g/L succinic acid was obtained from bread waste by solid-state fermentation with *Aspergillus awamori* and *Aspergillus oryzae*, which can produce complex enzymes containing a high amount of amylolytic and proteolytic enzymes, respectively.^51^

Another valuable product obtained from waste materials of foods with microbial fermentation is single-cell protein.^52^ The demand to formulate innovative and alternative proteinaceous food sources is increasing, because of concerns about population growth and the increasing number of hungry and chronically malnourished people. The most crucial step to respond to this demand is single-cell protein production.^53^ Single-cell protein, the extracted protein from microbial biomass like bacteria, yeast, algae, and fungi, can be used as a supplement protein source instead of conventional high-cost protein sources in the staple human diet to alleviate problems related to protein scarcity.^33,53,54^ Besides the nutritional benefits of using single-cell proteins in human or animal diet, another advantage is reducing the costs of final products during the formulation of food and fodder stocks, rich in protein, by using bioconversion products from wastes of agriculture and industry.^53^ As an example of innovative biotechnology, several species of insects have been employed to valorize the residual biomasses. The insects can incorporate the nutrients of organic wastes into their bodies. This ability of insects can reduce the amount of waste material, creating more valuable and homogeneous biomass.^55^ For example, the biotreatment of food waste by black soldier fly (*Hermetia illucens*) larvae provides volume reduction of the wastes and production of high-quality animal feed. It can recover, recycle, and valorize the food waste materials as constituents of animal feed and grass fertilizers.^56^ Biopolymers, other important products obtained from food wastes and byproducts, include a wide variety of products. These biopolymers are used in critical applications in different industries like medicine, cosmetics, pharmaceutical and food industries, water treatment, production and development of biosensors, industrial plastics, and clothing fabrics, because of their biodegradability, biofunctionality, biostability, and biocompatibility.^57^ Food waste is also utilized for bioplastic production includingpolyhydroxyalkanoates (PHA) and polyhydroxybutyrate (PHB) as organic polymers that can completely degrade into carbon dioxide and water within months after they are buried.^2^ Therefore, bioplastic production from food waste contributes to reducing both plastic waste and food waste.^58^

#### 4.1.2 Extraction Methods of Valuable Compounds from Agricultural Food Wastes/Byproducts

The biobased molecules, natural biopolymers, and phytochemicals can be obtained directly by extraction instead of synthesizing them from petroleum-based chemicals.^32^ Once these valuable biobased products are extracted, they can be considered high-value products such as food additives, nutraceuticals, therapeutics, and cosmetics.^33^ However, the economic feasibility of the extraction of the high-value components must be considered. In order to provide this economic feasibility, the desired components should be extracted by an appropriate method to obtain all of the valued components for full exploitation of the waste.^8^

The applied extraction techniques show differences based on the nature of the food matrix and the bioactive food ingredient, which will be extracted.^33^ Also, the extraction method used and the cellular matrix of the byproduct dominantly affect the recovery rate of a chemical entity.^59^ The extraction techniques can be divided into conventional and nonconventional.^60^ The conventional methods, consisting of solvent extraction, Soxhlet, maceration, and hydrodistillation, are characterized by temperature, agitation, and organic solvents like methanol, ethanol, and acetone.^33,60^ To maximize the resistance of the bioactive components, operating parameters including temperature, contact time, pH, particle size, solid-to-liquid ratio, and the stirring rate should be chosen appropriately. Longer extraction time, high volume of solvent use causing the generation of a high amount of toxic waste, and the need for the application of isolation or clarification technique as a final step for obtaining the extract without any solvent residues or impurities because of the use of toxic and expensive organic solvents are the disadvantages of these conventional techniques.^33,61^ The reduction of solvent consumption and extraction time, the improvement of extraction efficiency, and the use of greener solvents lead to more effective, cleaner, and greener modern or nonconventional techniques with decreased energy usage and organic solvent implementation are environmentally beneficial.^60,61^ Microwave-assisted, ultrasound-assisted, pressurized liquid, supercritical fluid, pulsed electric field-assisted, and enzyme-assisted extractions are highly examined novel thermal and nonthermal extraction techniques. Compared to conventional methods, the advanced extraction techniques listed above are more effective when their higher extraction efficiency, lower solvent consumption, lower extraction time, and energy cost are considered.^8,33,62,63^ Since the conventional solvents used in waste valorization have some disadvantages, because of their high price, high toxicity, and melting points, the search for a green and cost-effective technique has led to the emergence of deep eutectic solvents (DESs) and their bioanalogs, natural deep eutectic solvents (NADESs).^64,65^ The DESs and NADESs are considered environmentally friendly and novel solvents, which have a high capacity of dissolving biomasses to valorize food wastes effectively. They have been used to extract valuable components from wastes of vegetable oils, dairy, beverage products, and also from several natural raw materials such as lignocellulosic biomass, bark, wood, andalgae.^64^ For example, a simple, nonexpensive, and eco-friendly NADES has been designed to extract phenolic compounds from agro-food industrial byproducts. This eutectic solvent, which was prepared by combining lactic acid, glucose, and water, has been effectively applied to onion, olive, tomato, and pear byproducts, indicating the versatility of the technique.^66^ The usage of deep eutectic solvents can increase the interest in green extraction of food wastes to recover new high-quality products and functional ingredients in the food industry.^64^

As mentioned above, agricultural food wastes may be good sources of valuable compounds. Therefore, different extraction methods are widely investigated in the scientific community valorizing these beneficial compounds and reducing their harmful effects on the environment. Different types of extracted compounds from agricultural food wastes and byproducts by diverse extraction techniques applied in the last years are summarized in Table 1. The information about the extraction processes and their optimization can provide an excellent opportunity to design and improve innovative and functional products at the industrial level to valorize valuable compounds in agricultural food wastes.

**Table 1:** Examples of Extraction Methods and Extracted Valuable Compounds from Agricultural Food Wastes and Byproducts

agricultural food waste/byproduct	extraction method	optimal conditions of the extraction methods providing the best yield	extracted valuable compounds	ref(s)
kiwi juice pomace	microwave-assisted extraction with different conditions	optimal conditions for extraction at a microwave power of 400 W and pressure of 350 psi → solvent composition: 50% ethanol:water, solid-to-solvent ratio: 1:15 at 75 °C for 15 min	bioactive compounds based on **total phenolic content, flavan-3-ol content and ascorbic acid content** (high bioactive compound content and antioxidant potential of extract with optimal conditions)	(67)
			optimized extract (**total polyphenol content**: 4.8 ± 0.1 mg GAE g^–1^, **total flavonoid content**: 1.38 ± 0.01 mg CAT g^–1^, **ascorbic acid content**: 120.6 ± 0.5 mg 100 g^–1^)	
pistachio hard shells	extraction with different solvents/microwave-assisted extraction	optimal conditions for microwave-assisted extraction → using EtOH at 1000 W for 270 s	bioactive compounds based on **total phenolic content and total flavonoid content** (the highest yield (3.00% w/w) with microwave-assisted extraction at optimum conditions)	(68)
			the highest bioactive compounds in the microwave-assisted extract: **gallic acid, monogalloylglucose isomer, pentagalloylglucose, and kaempferol**	
			extract by microwave-assisted extraction at optimum conditions (**total phenolic content** (332 ± 11 mg GAE/g), **total flavonoid content** (376 ± 22 mg/CatEg), **antioxidant activity** by DPPH, TEAC, and ORAC (6.1 ± 0.9 μg/mL, 4001 ± 7.5 μmol TE/g, 879 ± 17 μmol TE/g, respectively)	
tomato processing waste	ultrasound-assisted extraction/conventional organic solvent extraction	optimal conditions for ultrasound-assisted extraction → solvent: hexane:acetone:ethanol (2:1:1 v/v/v) including 0.05% (w/v) butylated hydroxy toluene (BHT), solid liquid ratio (1:35 w/v) at 15 °C, 90 W for 30 or 15 min	**lycopene and β-carotene** (maximum lycopene and β-carotene yields with ultrasound-assisted extraction at 90 W for 30 and 15 min, respectively)	(69)
			**lycopene** (76.87 mg/kg dry weight) by ultrasound-assisted extraction at 90 W for 30 min	
			**β-carotene** (6.12 mg/kg dry weight) by ultrasound-assisted extraction at 90 W for 15 min	
pulp of hot pepper paste	ultrasound-assisted extraction/maceration extraction	optimal conditions for ultrasound-assisted extraction → 60% amplitude and 60 °C for 5 min	higher amount of **capsaicin and phenolic content** in a shorter time by ultrasound-assisted extraction	(70)
		optimal condition for maceration extraction for 8 h → 50 °C	content of the extract by maceration extraction at optimal conditions (**β-carotene content**: 192.85 mg β-carotene/100 g dry matter, **capsaicin content**: 299.49 μg capsaicin/g dry matter, **total phenolic content**: 277.23 mg GAE/kg dry matter, and **total antioxidant activity**: 170.59 μM TEAC/g dry matter)	
			content of the extract by ultrasound-assisted extraction at optimal conditions (**β-carotene content**: 230.544 mg β-carotene/100g dry matter, **capsaicin content**: 781.42 μg capsaicin/g dry matter, **total phenolic content**: 710.78 mg GAE/kg dry matter, and **total antioxidant activity**: 189.25 μmol TEAC/g dry matter)	
beetroot waste	pressurized liquid extraction (at 40 °C and 7.5, 10, and 12.5 MPa for 90 min, flow rate of 3 mL min^–1^)	optimal conditions for pressurized liquid extraction at 40 °C for 90 min → the mixture of ethanol:water 70/30 at 10 MPa	the highest global yield of extract from leaves (36.0% w/w) with pressurized liquid extraction under optimum conditions	(71)
			extract from leaves with pressurized liquid extraction at optimum conditions (**total phenolic content** (7 ± 1 mg_GAE_ g_extract_^–1^)	
			bioactive compounds (the most abundantly detected phenolic compounds: **ferulic acid, vitexin, and sinapaldehyde**)	
grape pomace	ultrasound-assisted extraction/microwave-assisted extraction	optimal conditions for microwave-assisted extraction → with 2% citric acid at 1000 W for 10 min	bioactive compounds (the recoveryof **anthocyanins → 45%**, **total phenolic content** (6.68 ± 0.05 mg_GAE_ g^–1^ (dry basis)), **total monomeric anthocyanins** (1.32 ± 0.03 mg malvidin-3,5-diglycoside g^–1^ (dry basis)), **antioxidant activity** by ABTS and DPPH (23.84 ± 0.57 μmol_TE_ g^–1^ (dry basis), and 33.27 ± 2.00 μmol_TE_ g^–1^ (dry basis), respectively with microwave-assisted extraction at optimum conditions)	(72)
sesame bran	enzyme-assisted extraction/ultrasound-assisted extraction/ultrasound-assisted enzymatic extraction	optimal conditions for ultrasound-assisted enzymatic extraction → pH (9.8), and 1.248 AU (Anson unit)/100 g enzyme concentration at 836 W, 43 °C for 98 min	**protein and antioxidant** compounds (**total phenolic contents** (3.82–6.03 mg GAE/g) **antioxidant capacity** based on DPPH (1.24–3.55 μmol TE/g), and **antioxidant capacity** based on ABTS (37.9–42.3 μmol TE/g) by ultrasound-assisted enzymatic extraction designs)	(73)
			the highest protein yield, total phenolic content, and antioxidant capacities with ultrasound-assisted enzymatic extraction under optimum conditions	
coffee husk	supercritical CO_2_ extraction	optimal conditions for supercritical CO_2_ extraction → solvent to raw material mass ratio: 197 kg CO_2_/kg husks at 373 K and 300 bar	**caffeine** (the extract yield (59%), the purity of caffeine (77%), and the caffeine yield (84%) with extraction under optimum conditions)	(74)
pumpkin seeds	ultrasound-assisted three-phase partitioning extraction	optimal conditions for ultrasound-assisted three-phase partitioning extraction → (NH_4_)_2_SO_4_ addition:30 g/100 mL, a *t*-butanol to slurry ratio of 1.0:1.0 (mL:mL), pH 5, and a duty cycle of 60% at 118 W for 20 min irradiation time	**oil, protein, and polysaccharide** (the optimal yields (39.79%, 14.30%, and 1.97%, respectively) with extraction under optimum conditions)	(75)
			fatty acid composition of extracted oil (**palmitic acid** (12.27%), **oleic acid** (32.35%), **linoleic acid** (48.58%), **saturated fatty acids** (18.73%), **monounsaturated fatty acids** (32.35%), and **polyunsaturated fatty acids** (48.91%))	
rice bran	conventional organic solvent-based Soxhlet extraction/subcritical CO_2_ Soxhlet extraction	optimal conditions for subcritical CO_2_ Soxhlet extraction → solvent-to-feed ratio: 24:1 at 68–70 bar and 27–29 °C	**Oil** (the yields (22% for conventional Soxhlet extraction and 13%–14.5% for subcritical CO_2_ Soxhlet extraction))	(76)
			nearly 10 times more thermolabile compounds, such as **tocopherols** (45.40–64.37 μg/g of oil), **tocotrienols** (157.89–198.31 μg/g of oil), and **oryzanols** (1.33–2.18 mg/g of oil), **lower** free fatty acid and peroxide values in extracted oil with subcritical CO_2_ Soxhlet extraction	
wheat bran	ultrasound-assisted enzymatic extraction	optimal conditions for ultrasound-assisted enzymatic extraction → raw material concentration: 50 g/L, enzyme dose: 4.5 g/L, at 180 W, 50 °C for 70 min	**Arabinoxylan** (the experimental yield (142.6 mg/g of wheat bran))	(77)
green plantain peels	wet extraction	optimal conditions for wet extraction → 5% w/v ascorbic acid concentration	**starch** (the average yield based on dry mass (29%) with nearly 70% purity)	(78)
			increase in starch yield with increase in antioxidant concentration whereas no significant effect of immersion time on final yield	
broccoli stalk	extraction with 0.1 M nitric acid	optimal conditions for extraction with 0.1 M nitric acid → liquid-to-solid ratio of 25 (v/w), 30 min	extracted **pectin** (the main neutral sugars:**rhamnose** and **galactose**, **homogalacturonan** and **rhamnogalacturonan I** substituted with **β-1,4-D-galactan** detected in extract based on analyses of nuclear magnetic resonance spectroscopy)	(79)
			75% **galacturonic acid** with a degree of methyl-esterification of 56%, and an acetyl content of 1.1% found in the pectin fraction of extract	
			pectic fraction of extract (yield 18%)	
watermelon rind	microwave-assisted extraction with different acid solutions (for 15 min at 39.9 W)	optimal conditions for microwave-assisted extraction → 1 N sulfuric acid for 15 min at 39.9 W	**pectin** (the highest yield (18%) with extraction under optimum conditions)	(80)
banana peels	extraction with 0.5 N hydrochloric acid/citric acid (at 90 °C for 1, 2, 3, 4 h)	optimal conditions for extraction → 0.5 N HCl at 90 °C for 4 h	**pectin** (the highest yield (17.05% dry basis) with extraction under optimum conditions)	(81)
			characteristics of extracted pectin from ripe banana peels using 0.5 N HCl for 3 h (moisture content (10.00%), ash (11.15%), **methoxyl content** (6.40%), **anhydrouronic acid** (57.32%), and **degree of esterification** (63.37%))	
			characteristics of extracted pectin from unripe banana peels using 0.5 N HCl for 3 h (moisture content (14.13%), ash (13.83%), **methoxyl content** (5.25%), **anhydrouronic acid** (39.68%), and **degree of esterification** (75.03%))	
orange peels	hot-water extraction/rapid solid liquid dynamic (RSLD) extraction/microwave-assisted extraction	optimal conditions for acidic hot-water extraction → liquid-to-solid ratio (20), pH (1.5) at 70 °C for 60 min	**pectin** (the highest yield (21%) by acidic hot-water extraction under optimum conditions)	(82)
			extracted pectin by acidic hot-water extraction at optimum conditions (**esterification degree** (82.5), morphology (stressed surface), thermogravimetric properties (the temperature at which the 50% of the mass loss occurs: 304 °C, the temperature that corresponds to the maximum decomposition rate: 243 °C, the total mass loss at 650 °C: 74%))	
kiwi seeds	conventional Soxhlet extraction/microwave-assisted extraction/supercritical CO_2_ extraction/ultrasound assisted extraction/microwave integrated Soxhlet extraction	optimal conditions for ultrasound-assisted extraction → 10 mg seed powder in 400 mL *n*-hexane at 50 °C, 80 W for 30 min	**oil** (the highest yield (28.9 oil/seed wt %) with ultrasound-assisted extraction at optimum conditions)	(83)
			composition of extracted oil by ultrasound-assisted extraction at optimum conditions (**total saturated fatty acids** (8.94%), **total monounsaturated fatty acids** (14.79%), and **total polyunsaturated fatty acids** (76.27%))	
			the presence of off-flavors in oil samples with conventional Soxhlet extraction and ultrasound-assisted extraction	
lemon peels	sequential extraction with a combination of microwave-assisted extraction and hydrodistillation and microwave-assisted extraction	optimal conditions for combination of microwave-assisted extraction and hydrodistillation → water-to-solid ratio: 0.3 mL/g, 1st step (irradiation power: 1.2 W/g for 5 min) and 2nd step (irradiation power: 0.7 W for 15 min)	**essential oil** extracted by a combination of microwave-assisted extraction and hydrodistillation: **limonene** (65.082 wt %), **β-pinene** (14.517 wt %), and **γ-terpinene** (9.743 wt %)	(84)
			essential oil (potent inhibition against *E. coli* and *S. aureus* bacteria)	
			**pigment** extracted by microwave-assisted extraction: **eriocitrin** (1.096 wt %), **diosmin** (1.645 wt %), and **hesperidin** (0.529 wt %) in the initial extract	
		optimal conditions for microwave-assisted extraction → 80% (v/v) ethanol, 80 °C and 50 min, with a liquid-to-solid ratio of 1:10		
			yield of essential oil and pigment: ∼2 and 6 wt %, respectively	
lemon peels	conventional Soxhlet extraction/high-pressure–high-temperature extraction	optimal conditions for high-pressure–high-temperature extraction → at 150 °C for 30 min and matrix solvent ratio: 1:15	d-limonene (the highest yield (3.56%) with high-pressure–high-temperature extraction under optimal conditions)	(85)
citrus peels	conventional hydrodistillation/microwave-assisted hydrodistillation	optimal conditions for microwave-assisted hydrodistillation at constant pressure (300 mbar), solid-to-liquid ratio (1:1.5) → 1st step (irradiated with 785 W for 5 min) and 2nd step (irradiated with 250 W for 15 min)	**essential oil** from *Navel Navelate* oranges (**monoterpenes**, **oxygenated monoterpenes**, and **sesquiterpenes)** chemical composition of essential oil from *Navel Navelate* oranges by conventional hydrodistillation **(monoterpenes** (98.56%) and **oxygenated monoterpenes** (0.14%)) chemical composition of essential oil from *Navel Navelate* oranges by microwave assisted hydrodistillation (**monoterpenes** (99.34%), **oxygenated monoterpenes** (0.14%), and **sesquiterpenes** (0.01%)) the most abundant chemical of essential oil from *Navel Navelate* oranges: d-**limonene** (96.75% for conventional hydrodistillation and 97.38% for microwave assisted hydrodistillation) the yield based on dry basis for *Navel Navelate* oranges (1.8% with microwave assisted hydrodistillation and 1.7% with conventional hydrodistillation)	(86)

### 4.2 Improving the Stability and Bioavailability/Accessibility of Bioactive Compounds Derived from Agricultural Food Wastes/Byproducts

The valorization of biomass by conventional methods such as solid–liquid extraction after maceration and novel and green methods such as extraction with sonication, supercritical fluids, microwaves, and pulsed electric fields recently attracted attention, because of their high phytochemical content, which provides antioxidant, anti-inflammatory, and antibacterial properties leading to potential health benefits.^87,88^ For instance, these health-promoting bioactive compounds in fruit and vegetable wastes play an essential role in their anticancer, antimutagenic, antiviral, antioxidant, antitumor activities and their ability to reduce the risks of cardiometabolic diseases. Also, recent studies focusing on phytochemicals state that the high antioxidant properties of polyphenols from plant sources, including skins, seeds, pulp, or pomace, make polyphenols one of the primary phytochemicals important for health aspects.^89^

Although the demand and consumer acceptance for natural antioxidants in the food industry are constantly increasing to prevent harmful chemical additives and inhibit the oxidation processes in the final product, there are several problems with the utilization of natural antioxidants in various food products.^87,88^ Most of them are challenged by chemical degradation in foods and the gastrointestinal tract (GIT), reducing their bioavailability and bioactivity.^87^ In addition, many of them have a poor solubility problem, leading to a restriction of their direct incorporation in some foods, and many of them struggle with sensitivity to oxygen, light, heat, enzymes, salts, and acid or alkaline media, causing losses in their beneficial effects and activity.^88^ In recent years, scientists have performed extensive research to increase the amount and bioavailability of the components obtained from food wastes/byproducts and improve their stability during food processing and gastrointestinal digestion. The most common novel techniques to overcome these limitations are applied by nanotechnological approaches such as encapsulation, nanoemulsion, and biotechnological processes such as fermentation and enzyme use.

Nanotechnology can be defined as a technique developed to study, design, create, synthesize, apply and manipulate materials, devices, and functional systems by using nanoscale control of materials.^90^ Recently, microencapsulation and nanoencapsulation techniques have been applied for biomass wastes to find solutions to previously explained problems by increasing their stability, solubility, and bioavailability.^87,88^ Within the scope of nanoencapsulation, edible nanoparticles, with a maximum particle size of 500 nm and composed of proteins, carbohydrates, lipids, phospholipids, or surfactants, trap the phytochemicals inside with different applications like spray drying, freeze-drying, coacervation, crystallization, molecular encapsulation, extrusion, and electrostatic extrusion to increase the efficiency and management of the phytochemicals by enhancing their stability, bioavailability, bioactivity, and dispersibility.^87,89^

Since nanotechnological approaches provide higher stability, bioaccessibility, and bioavailability for bioactive compounds, they can potentially lead to innovative and functional products, especially in the food and pharmaceutical industries. The studies in the last five years about the application of different encapsulation techniques and their effects on the extracts derived from agricultural food wastes or byproducts are summarized in Table 2.

**Table 2:** Encapsulation Methods and Their Effects for Extracted Valuable Compounds from Agricultural Food Wastes

extract	encapsulation method	wall material	encapsulation efficiency	effects of encapsulation	references
carotenoids from carrot processing waste	spray drying/freeze-drying	whey protein/maltodextrin/Inulin	freeze-drying with pure whey protein (63.69 g/100 g)	freeze-dried encapsulate (**best** hygroscopicity, oxidative stability, antioxidant capacity, and color properties)	(93)
			spray drying with the mixture of 71 g/100 g whey protein and 29 g/100 g inulin (53.78 g/100 g)		
			spray-dried encapsulate (**lowest** water activity, moisture content, and particle size)		
carotenoids from tomato peels	electrospinning	Zein fibers	>90%	nanoencapsulation process (**an increase** of 11-fold antioxidant activity)	(94)
				encapsulated extract (**better retention** of lycopene and antioxidant activity during 14-days storage time than nonencapsulated extract)	
phenolics and carotenoids from red pepper waste	spray drying/freeze-drying	whey protein	slightly better results for freeze-drying than spray drying method	encapsulation process (**a protective effect** against pH changes and enzymatic activities along with digestion and **increase** in bioaccessibility in the gut) (**an efficient method** for improvements in nutrition, color, and bioactive properties)	(95)
phenolic compounds from winemaking waste	extrusion	alginate and chitosan	between 55% and 79%	encapsulation process (**retention** of chemical stability and biological activities)	(96)
			more suitable and efficient wall material (the mixture of alginate and chitosan at concentrations of 1% w/v and 3% w/v, respectively, and encapsulating about 80% of the extracts)		
phenolic compounds from cornsilk	spray drying/freeze-drying/microwave drying	maltodextrin	freeze-drying (99.84%)	freeze-drying with 100% maltodextrin (**the highest recovery** of phenolic compounds and **the highest retention** of antioxidant activity)	(97)
			microwave drying (99.83%)		
			spray drying (99.65%)		
phenolic compounds from unused chokeberries	spray drying/co-crystallization/ionic gelation	maltodextrin/skim milk powder/whey protein/alginate/sucrose syrup	spray drying (99.6%)	spray-dried encapsulate (**higher** phenolic content and **better** stability, but **lower** radical scavenging activity than encapsulates by other methods)	(98)
			co-crystallization (96.3%)		
			ionic gelation (94.2%)		
encapsulated extracts (**reduction** of 6.07%, 24.75%, and 52.97% was observed in total phenolic content by spray drying, co-crystallization, and ionic gelation, respectively, during storage time at 5 °C)				encapsulated extracts (**reduction** of 6.07%, 24.75%, and 52.97% was observed in total phenolic content by spray drying, co-crystallization, and ionic gelation, respectively, during storage time at 5 °C)	
phenolic compounds from golden apple and red grape pomace	nanoemulsification	chitosan/soy protein	nanoemulsification with soy protein (95%)	encapsulation process (**improvement** of antioxidant activity of phenolic extracts)	(99)
			nanoemulsification with chitosan (75%)		
carotenoids and phenolic compounds from sweet potato peels	spray drying/freeze-drying	whey protein	spray drying for carotenoids and phenolics (60.0% and 61.9%, respectively)	encapsulation process (**better quality** of encapsulates based on water activity, moisture content, hygroscopicity, and encapsulation efficiency of phenolics by freeze-drying and **smaller** particle size, **better** flow properties, and encapsulation efficiency of carotenoids by spray drying)	(100)
			freeze-drying for carotenoids and phenolics (9.34% and 64.3%, respectively)		
				spray drying (**retention** of carotenoid and phenolic compounds and **prolonged shelf life** under light and dark storage conditions)	
blueberry residue	ionotropic gelation	sodium alginate	-	encapsulation process (67.01% **retention** of the phenolic compounds and 68.2% **release of phenolics** (120 min) after in vitro dissolution)	(101)
bioactive compounds from lemon byproducts	spray drying/freeze-drying	maltodextrin/soybean protein/ ι-carrageenan	freeze-drying with the mixture of maltodextrin and soybean protein (more efficient technique than spray drying)	encapsulation process (**the highest retention** of total phenolic content, total flavonoid content, and ferric ion reducing antioxidant power by freeze-drying with the mixture of maltodextrin and soybean protein)	(102)
				freeze-dried encapsulate (**lower moisture content and water activity** than those produced by spray-drying)	
bioactive compounds from beetroot pomace	freeze-drying	soy protein	86.14%	encapsulation process (**retention** of polyphenols (76.67%), betalain pigments, betacyanins (17.77%), and betaxanthins (17.72%) during storage time (three months) at room temperature)	(103)
				encapsulated extracts (**higher release** of polyphenolic compounds in simulated intestinal fluid than in gastric fluid during in vitro digestion)	

In addition to the encapsulation methods provided in Table 2, emulsion-based systems can also be applied to valorize and improve compounds derived from food waste efficiency and stability. For example, extracts rich in mango peel phenolics were encapsulated in water-in-oil-in-water emulsions with different surfactants (Tween 20, Tween 80, and lecithin). Water-in-oil-in-water emulsion with Tween 20 had the highest encapsulation efficiency (98.65% ± 1.14%). In contrast, the best physical and encapsulation stability within the storage period was obtained in emulsions with Tween 80. These results showed that the application of efficient and stable emulsion-based systems with suitable surfactants could successfully encapsulate phenolic compounds.^91^ Also, another study indicated that mixing excipient emulsion (oil-in-water) prepared by microfluidics and tomato pomace has increased the total phenolic content of tomato pomace and lycopene bioaccessibility in tomato pomace.^92^ The study of Zhu et al. indicated that the nanoencapsulation systems are commercially used in several countries worldwide. The NanoCeuticals Supplements—RBC Life Sciences in Germany, consisting of NANOCLUSTERS technology, use nanosized powders to improve organoleptic properties and increase bioavailability. Super Nano Green Tea, which consists of nanometric particles (200 nm) with increased bioavailability, and Nano-Selenium Rich Black Tea with improved selenium bioavailability, can be given as examples of commercial nanoencapsulation applications in China. Furthermore, as another example, Nano Gold (NGT) edible gold is produced with physical methods in Taiwan as gold nanometric particles, with diameters of 0.5100 nm.^90^ Although some countries commercially use nanotechnological approaches, some regulations and further in vivo studies to determine their long-term effects are necessary for their extensive usage.

Biotechnological approaches, the most well-known of which is fermentation, have been used to valorize the food wastes and byproducts by converting them into functional components.^104^ Therefore, in addition to the above-mentioned nanotechnological approaches, numerous studies focus on fermentation to increase the content of bioactive compounds and their bioavailability. Fermentation, which appears as one of the oldest processes used to convert products to value-added products, occurs by breaking down the organic compounds to obtain energy through anaerobic metabolism.^105,106^ The fermentation process is highly preferred in scientific and industrial fields, since it substantially satisfies the need to limit the amount of waste produced. It consumes less energy, generates a small amount of water, and has a low cost.^106^ Three types of fermentation processes, solid-state, submerged, and liquid fermentation are applied, depending on the product type. Solid-state and submerged fermentation are the two most commonly used processes in novel research and industry to obtain bioactive compounds.^105^ Recently, the fermentation process, in which desired bioactive compounds such as antioxidants are produced, is becoming more prevalent in scientific and industrial fields, regarding nutritional and health issues.^107^ Different examples of the effects of fermentation (especially solid-state fermentation) on the bioactive compounds from agricultural food wastes/byproducts are summarized in Table 3.

**Table 3:** Effects of Fermentation on the Bioactive Compounds from Agricultural Food Waste/Byproducts

agricultural food waste/byproduct	fermentation method	microorganisms	effects of fermentation	reference
mango seeds	solid-state fermentation	*Aspergillus niger*	**mobilization** of polyphenolic compounds	(110)
			**improvement** of nutraceutical properties	
			practical method for **releasing** the bound phenolics	
apricot pomace	solid-state fermentation	*Aspergillus niger* and *Rhizopus oligosporus*	**increase** of total phenolic and flavonoid contents (over 70% for total phenolics and 38% for total flavonoids with *R. oligosporus*, and more than 30% for total phenolics and 12% for total flavonoids with *A. niger*)	(111)
			**improvement** of free radical scavenging capacities	
plum fruit (*Prunus domestica* L.) byproducts	solid-state fermentation	*Aspergillus niger* and *Rhizopus oligosporus*	**significant increase** of total phenolic and antioxidant levels (for total phenolic content: higher than 30% with *R. oligosporus* and higher than 21% with *A. niger*)	(112)
			**achievement** of higher lipid recovery from plum kernels	
			**enrichment** of polar lipids with n-3 polyunsaturated fatty acids	
spent coffee grounds	solid-state fermentation	*Bacillus clausii*	**increase** of total phenolic and flavonoid contents and antioxidant capacity (36%, 13%, and 15%, respectively)	(113)
			**improvement** of antimicrobial activity against gram (+) and gram (−) bacteria	
spent coffee grounds	fermentation	*Bacillus clausii*	**increase** of total proteins, soluble proteins, and protein hydrolysates amounts (2.7-, 2.2-, and 1.2-fold, respectively)	(114)
tomato seed meal extract	fermentation	*Lactobacillus plantarum*	**reduction** of crude and soluble proteins contents (18.44% and 68.99%, respectively) due to *L. plantarum* growth on the substrate after 24 h	(115)
			**increase** of radical scavenging activity after 24 h (87%) due to different bioactive peptides production	
			**significant reduction** of total amino acids (specifically glutamic acid and aspartic acid) concentration	
			**improvement** of new amides and aromatic compounds formation	
peanut press cake	solid-state fermentation	*Aspergillus awamori*	**improvement** of phenolic and antioxidant properties	(116)
			**improvement** of functional properties (apart from bulk density)	
			**improvement** of morphological characteristics and mineral content	
Jussara pulp	*Lactobacillus* fermentation	*Lactobacillus* and *Bifidobacterium* strains	**increase** in antioxidant activity	(117)
			**more extensive changing** of jussara anthocyanins by *Lactobacillus deubruekii*	
			the main bioconversion products from anthocyanins → protocatechuic acid	
rice bran	solid-state fermentation	*Rizhopus oryzae*	**increase** of phenolic compound content (more than 2-fold)	(118)
			**the highest increase** of phenolic acid → gallic and ferulic acid	
			phenolic extract from fermented rice bran → **inhibition** of the peroxidase enzyme	

Other innovative biotechnological approaches to valorize high-value ingredients from fruit and vegetable wastes are present along with fermentation. They are primarily used in drug and functional food formulation as natural bioactive compounds.^108^ For example, enzyme complexes can hydrolyze materials, releasing desired products in a wide range of food waste. The studies have focused on using several enzyme complexes, mainly α-amylase, cellulases, xylanases, pectinases, proteases, chitinase, according to the composition of a specific substance. The wide range of products derived from food wastes with the help of these enzyme complexes include antioxidants, protein hydrolysates, pigments, oligosaccharides, growth-promoting substrates, etc.^104^ For example, the effects of enzymatic treatment using only tannase, pectinase, and cellulase, or a mixture of these three enzymes on the phenolic compounds of grape pomace were investigated. The results showed that grape pomace’s total polyphenol content and antioxidant activity were increased with enzymatic treatments, which released phenolic acids and aglycones from grape pomace, and tannase^109^ obtained the greatest hydrolytic efficacy. It is advantageous to prefer biotechnological approaches, especially fermentation, for food waste valorization at an industrial level, because of their low energy requirement and low cost. In addition, biotechnological approaches are good options for food waste valorization to design innovative functional foods, because of their nutritional and health benefits. The food wastes that cannot be used directly in food products can be integrated with nanotechnological and biotechnological approaches.

## 5 Use of Agricultural Food Wastes/Byproducts and Their Extracts As Additives in Food Products

The market currently deals with discussions on synthetic additives without reaching a consensus.^90^ This situation leads the food industry to search for natural food additives produced using novel, natural, and economical protein sources, dietary fiber, flavoring agents, colorants, antioxidants, and antimicrobials.^90,119^ Since the environmental problems can be reduced, and food additives or supplements with high economic and nutritional value can be utilized, byproducts as a source for natural food additives emerge as a good option. Therefore, the companies can choose to transform these valuable byproducts into a value-added product which allows them to decrease their treatment cost, create additional profit, and thus make them more competitive in the market.^119^ Also, some biotechnological processes are available to diminish the antinutritional factors found in some of the byproducts and, therefore, allow them to be a source of food additives or be part of a balanced food formulation. Although the local communities in poor regions utilize their residues and byproducts by using novel food formulations, most small traders in those regions dispose of their wastes to the environment. Therefore, the Environmental Protection Agency (EPA) determined the order of priority to ensure that the surplus is used to feed hungry people first and then animals. The order of priority indicated that the rest of the surplus should be utilized in industrial uses. If they cannot be used, they should be composted and incinerated.^120^

With the contribution of food waste as natural additives into food products to provide nutrients and bioactive compounds, the sensory properties and consumer acceptance are also essential criteria for food products.^120^ For example, two different flavors—pineapple and white chocolate—are used to produce the jelly samples with the collagen extracted from chicken feet. This study demonstrated that both products have good sensory acceptance by tasters, and consumers can willingly consume them.^121^ However, another study on the acceptability of cookies enriched with antioxidant fiber using a blueberry pomace byproduct was conducted. The tasters were given a reference vanilla cookie and a new cookie elaborated with blueberry pomace. Although they liked the labels of the new cookie before tasting it, they did not find it better than the reference cookie.^122^ Several studies on the use of agricultural wastes in food formulations and their effects on the final products are listed in Table 4.

**Table 4:** Agricultural Food Wastes and by-Products As Natural Food Additives for Different Types of Food Products

functional additives	food product	final product quality	reference
encapsulated bioactives from red pepper waste	yogurt	**retention** of carotenoid (71.43%) and polyphenols (increase up to 123.73%) and higher pigment retention during 21 days of storage time	(125)
		**higher** sensory and general acceptance due to **better** color, appearance, and flavor	
encapsulated bioactives from grape skin	whole wheat cocoa biscuits (with 1.2, 2.3, and 3.5% addition of encapsulated extract on dough weight)	**increase** of phenolic content (up to 134%) and antioxidant capacity (up to 244%)	(126)
		reduction of phenolic content (16%) during cooking	
		**change** of color and not relevant positive impact on oxidative stability	
		encapsulated extract addition level (**significant influence** on the sensory acceptance)	
free and encapsulated phenolic extract from olive leaf	full-fat mayonnaise (∼80% oil)	addition of both types of extract (**improvement** of dispersion degree of the sample, and causing **lower** spreadability, **higher** salty and bitter taste)	(127)
		addition of encapsulated extract (**improvement** of physical properties)	
		enriched mayonnaise with free or encapsulated extract (**lower** overall acceptance)	
encapsulated black mulberry waste extract	dark chocolate	chitosan coated liposomal powders (**better** anthocyanin protection than spray-dried extract)	(128)
		encapsulation in liposomes (**improvement** of in vitro bioaccessibility of anthocyanins)	
		**maximum fortification** with encapsulated anthocyanins (76.8% based on conching temperature and pH)	
onion skin powder	bread	**enhancement** of bioaccessible lipid oxidation preventers and compounds having chelating and antioxidant abilities	(129)
		**higher** antioxidant activity	
		2%–3% of onionskin powder addition (**significant development** of the antioxidant capabilities)	
		up to 3% of onionskin powder addition instead of wheat flour (**satisfactory** consumers’ acceptance)	
lettuce waste flour	bread	**reduction** of dough leavening capacity, and increase of bread moisture and firmness, phenolic content (up to 3.4 g GAE kg^–1^), and antioxidant capacity (200%)	(130)
		addition of lettuce waste flour (at least 170 g kg^–1^) (**improved** fiber content (>30 g kg^–1^), **reduction** of yeast odor and flavor intensity, and **increase** of silage and herbaceous scent and flavor, dried fruit flavor, acid, and sour taste)	
		enriched bread with 170 and 575 g kg^–1^ addition of lettuce flour (**comparable sensory properties and consumer acceptability** with commercial wholemeal bread containing similar rye bran content)	
olive paste flour	durum wheat spaghetti	the **best addition amount** of olive paste flour for enrichment (10% (w/w))	(131)
		addition of 10% olive paste flour (an **increase** of total polyphenol content [from 82.39 μg/g DW to 245.08 μg/g DW], **15 times higher** in apigenin, luteolin, and quercetin levels)	
		addition of transglutaminase (0.6%) to enriched pasta with 10% olive paste flour (**increase** of overall pasta quality)	
		enriched spaghetti with 10% olive paste flour and 0.6% transglutaminase (**very similar quality** with the control sample for uncooked and cooked spaghetti)	
carrot pomace powder and dushab (a traditional grape juice concentrate)	cakes	addition of carrot pomace powder and dushab (**reduction** of specific volume of cakes and **increase** of moisture content, color difference, browning index, firmness, and **reduction** of cohesiveness with increase of added amount)	(132)
		**increase** of carrot pomace powder addition (**increase** of chewiness and gumminess)	
		addition of dushab (**not effective** on chewiness and gumminess, and **decrease** of cake springiness)	
grape residue flour	grape ice cream	addition of 2% of grape residue flour (**increase** of protein, lipids, ash, dietary fiber, total energy value, reducing sugars, total phenolics, flavonoids, flavonols, and anthocyanins, and **higher** radical scavenging ability) (satisfactory sensory attributes and the best formulation among other ice creams containing grape residue flour in different levels)	(133)
apple pomace powder	stirred-type yogurt	addition of apple pomace powder (2%–3%) (**alteration** on the structure of stirred yogurt)	(134)
		apple pomace powder addition into the diluted yogurt system (**potential stabilization** of acid drink and **reduction** of protein aggregates sedimentation)	
coffee pulp extract	probiotic beverage with or without kefir culture	probiotic beverage from coffee pulp extract by steam pretreatment with kefir culture (**improvement** of phytochemicals, physicochemical properties, and **enhancement** of organoleptic properties, the **best** overall acceptance according to the control beverage without kefir cultures)	(135)

Furthermore, the industry can have economic gains, the nutritional problems can be diminished, the environmental implications generating mismanagement of waste can be reduced, and some beneficial health effects can be produced if the food waste and byproducts are used adequately as sources of food additives. Recently, the industries are investigating innovative methods to achieve zero waste, which refers to generated waste as raw material for new products and applications. The Millennium Development Goals, the upcoming Sustainable Development Goals, and the Zero Hunger Challenge of the United Nations can be directly affected by these innovative methods designed to obtain zero waste.^120^ For example, the company GEA is running the Pro-Enrich project, which aims to investigate new approaches to valorize fruit and vegetable residues as functional proteins, phenols with antioxidant and antimicrobial properties, dietary fibers, and pigments in various applications. The trend to extract plant-based functional proteins and other valuable compounds from plants has increased due to environmental and economic issues of animal-based applications. Within this project’s scope, the excess of biomass generated after processing fruits and vegetables is provided by suppliers from various parts of the supply chain from the EU and the world. Pro-Enrich’s project focuses on new ways to extract valuable compounds while protecting their purity, functionality, and quality with energy-efficient and cost-effective methods. These valuable compounds extracted from the tomato residues, citrus fruit residues, olive pomace, olive mill wastewater, and rapeseed (canola) meal/press-cake are used in several industries such as dietary supplements, pet food, cosmetics, food ingredients, and adhesives.^123^ Another project conducted by the Food Innovation Centre of CSIRO (Commonwealth Scientific and Industrial Research Organization), an Australian government agency, focused on developing a new method to valorize biomass as value-added ingredients and food products. The project investigated a new process to stabilize the apple pomace, which was chosen as a model food source, to protect it from physical, chemical, and microbial degradation. The results showed that this process helps create a functional and nutritious ingredient. Therefore, it can be used for other fruits, vegetables, and horticultural products such as broccoli, carrots, olives, and grapes.^124^

## 6 Limitations and Future Perspectives

Food waste is considered a nutritional, functional, and nutraceutical raw material that can be used in various applications in food formulations, making it a potential solution for economic, social, and environmental problems. They can be used directly as food components or proteins, lipids, vitamins, fibers, starch, minerals, and antioxidants. Other biomolecules within the food waste can be physically or chemically extracted and used as nutritional and functional components. To maintain the physicochemical and microbiological stability of biomaterials during the valorization of these food wastes, the application of the unitary drying operation is necessary to prevent microbial risks. Thus, the governments should support installing the infrastructure and technology, which make the utilization of food wastes and byproducts in the production and storage areas possible.^120^ On the other hand, eliminating other risks, including toxic materials and antinutritional factors, should also be considered.

Another critical limitation about using food wastes/byproducts as functional food ingredients for the development and design of new food products is the sensory quality of the food materials and consumer acceptance. The use of food wastes/byproducts as functional ingredients in foods produced at the industrial level is limited compared to their use as biofuel in the industry. Therefore, more-detailed studies about the formulation of new functional foods are needed to achieve higher quality and consumer acceptance. In addition, the companies should try to valorize their food wastes and byproducts by integrating them into new industrial products. Indeed, changes in the processing steps and designs may be considered, for example, to reintegrate the wastes of their products to the original food at an industrial scale (i.e., tomato skin and seeds may be processed and restored into tomato paste/sauce). On the other hand, probably the intermediary companies (waste brokers) who collect wastes and direct them to specific points for processing into new products/components will increase in number in the future, which will be beneficial from the waste valorization point of view.
